# MiR-129 triggers autophagic flux by regulating a novel Notch-1/ E2F7/Beclin-1 axis to impair the viability of human malignant glioma cells

**DOI:** 10.18632/oncotarget.7003

**Published:** 2016-01-25

**Authors:** Xiong Chen, Yingying Zhang, Yingying Shi, Haiwei Lian, Huilin Tu, Song Han, Jun Yin, Biwen Peng, Beiyan Zhou, Xiaohua He, Wanhong Liu

**Affiliations:** ^1^ School of Basic Medical Sciences, Wuhan University, Wuhan 430071, China; ^2^ Hubei Provincial Key Laboratory of Developmentally Originated Disease, Wuhan 430071, China; ^3^ Hubei Province Key Laboratory of Allergy and Immunology, Wuhan 430071, China; ^4^ Department of Neurosurgery, Wuhan University Renmin Hospital, Wuhan 430060, China; ^5^ Department of Veterinary Physiology and Pharmacology, College of Veterinary Medicine and Biomedical Sciences, Texas A & M University, Texas 77843, USA

**Keywords:** miR-129, E2F7, Notch-1, autophagy, glioma

## Abstract

Abnormalities of autophagy have been implicated in an increasing number of human cancers, including glioma. To date, there is a wealth of evidence indicating that microRNAs (miRNAs) contribute significantly to autophagy in a variety of cancers. Previous studies have suggested that miR-129 functioned as an important inhibitor of the cell cycle and could promote the apoptosis of many cancer cell lines *in vitro*. Here, we reported that miR-129 acted as a potent inducer of autophagy. Forced expression of miR-129 could induce autophagic flux by targetedly suppressing Notch-1 in glioma cells. The autophagy induced by miR-129 could restrain the activity of mammalian target of rapamycin (mTOR) and upregulate Beclin-1. Moreover, we demonstrated that E2F transcription factor 7 (E2F7) could also trigger autophagic flux by upregulating Beclin-1 and mediating miR-129-induced autophagy. Additionally, knockdown of Notch-1 could upregulate the expression of E2F7, whereas downregulation of E2F7 alleviated shNotch-1-induced autophagic flux. In particular, knockdown of endogenous Beclin-1 could effectively reduce autophagic flux stimulated by miR-129 and E2F7. Interestingly, upon attenuation of miR-129- or E2F7-triggered autophagic flux rescued cell viability suppressed by them. More importantly, intratumoral injection of pHAGE-miR-129 lentivirus in a nude mouse xenograft model significantly restrained tumor growth and triggered autophagy. In conclusion, these findings identify a new function for miR-129 as a potent inducer of autophagy through a novel Notch-1/E2F7/Beclin-1 axis in glioma.

## INTRODUCTION

Autophagy is a cellular self-cannibalization process that involves sequestration of cytoplasmic constituents into double membrane vesicles called autophagosomes, followed by degradation via the lysosomal pathway [1]. To date, there is an increasing body of evidence to link autophagy and cancer, including glioma [2–4]. MiRNA is a small non-coding RNA consisting of 18–25 nucleotides, which regulates the expression of target genes by post-transcriptional suppression through interaction with the 3′UTR of the target mRNA [5]. miRNAs have been found to regulate diverse cellular processes, including cell proliferation [6], apoptosis [7], senescence [8] and differentiation [9]. Recently, the contributions of autophagy mediated by miRNA have become a rising concern in the field of cancer research [10–13].

MiR-129 is transcribed from two genes, miR-129-1 and miR-129-2 [14]. The ability of miR-129 to inhibit proliferation and promote apoptosis has been demonstrated in a variety of tumor cell lines [15–17]. Nevertheless, to our knowledge, the influence of miR-129 on autophagy has not been reported.

Notch-1 is critical in cell fate decisions in the developing nervous system [18] and glioma cells [19–21]. Moreover, Zou et al. reported that the mTOR/STAT3/Notch-1 pathway mediated oroxylin A-induced autophagic cell death in glioma cells [22]. However, it is unclear whether Beclin-1, an autophagic gene encoded by *BECN1*, is involved in Notch-1 inhibition-induced autophagy.

E2Fs are transcription factors that are well known for their involvement in the regulation of cell cycle progression [23]. Moreover, it has been shown that downregulation of E2F1 led to increased autophagy in glioma cells [24]. Overexpression of E2F1-E2F5 in serum-starved T98G cells promoted Beclin-1 expression [25]. Nevertheless, the role of E2F7, an atypical E2F family member, in autophagy is unclear.

In this study, we demonstrated that hsa-miR-129-5p, hereafter referred to as miR-129 unless particularly stated, could induce autophagy by targetedly suppressing Notch-1 in glioma cells. The mTOR signaling pathway and Beclin-1 were involved in autophagy induced by miR-129. Moreover, overexpression of E2F7 was shown to be capable of inducing autophagy by upregulating Beclin-1. Interestingly, enforced expression of miR-129 or inhibition of Notch-1 increased the expression of E2F7 in glioma cells. Finally, suppression of miR-129- or E2F7-induced autophagy by knockdown of Beclin-1 or knockdown of Atg5 restored cell viability. These findings provide new insights into the antitumor effects of miR-129 and may contribute to the application of miR-129 for glioma therapy.

## RESULTS

### Overexpression of miR-129 induced autophagy in glioma cells

**Figure 1:**
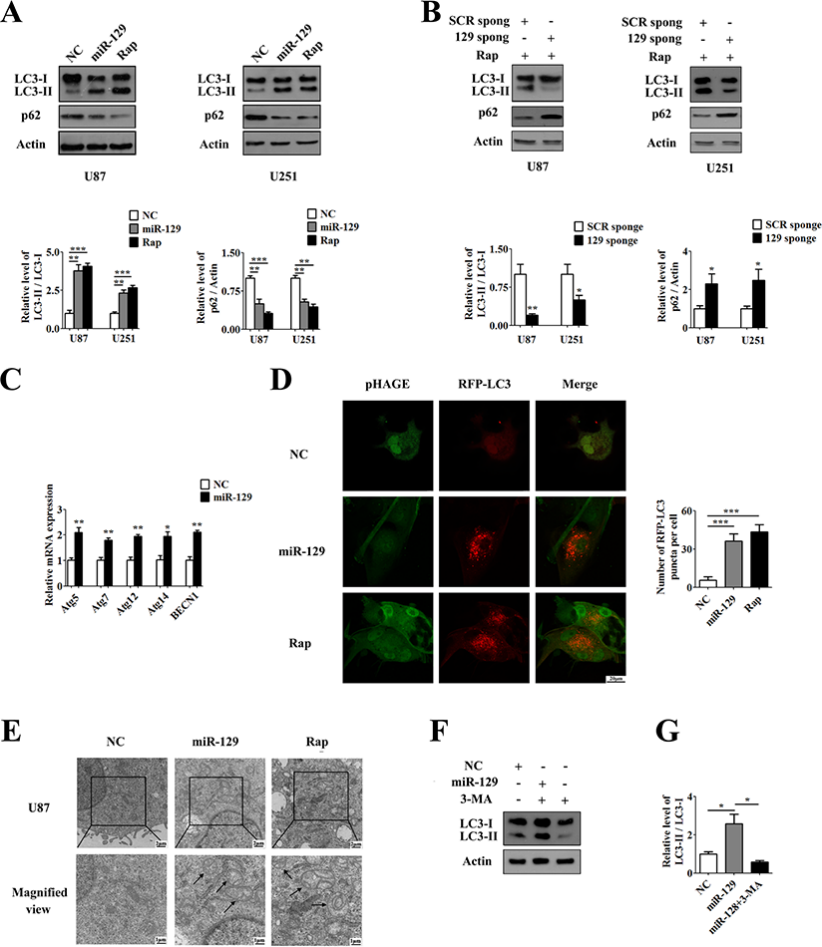
Overexpression of miR-129 induces autophagy in human glioma cells (**A**) MiR-129 induced LC3 conversion and p62 degradation. Cells were infected with Lv-NC or Lv-miR-129 for 96 hours or treated with 100 nM rapamycin (Rap) for 24 hours and then analyzed by Western blot. The LC3-II/LC3-I or p62/Actin ratios from immunoblots were measured by Image J densitometric analysis (mean ± SEM of independent experiments, *n* = 5, **P < 0.01, ***P < 0.001). (**B**) After transfection with SCR sponge or miR-129 sponge vectors for 48 hours, U87 or U251 cells were treated with 100 nM Rap for an additional 24 hours. Knockdown of miR-129 inhibited Rap-induced LC3-I to LC3-II conversion and p62 degradation. (mean ± SEM of independent experiments, *n* = 3, *P < 0.05, **P < 0.01). (**C**) qPCR analysis of mRNA expression levels of ATGs. U87 cells were infected with Lv-NC or Lv-miR-129 for 72 hours and harvested for qPCR. (mean ± SEM of independent experiments, *n* = 3, *P < 0.05, **P < 0.01, Student's 2-tailed *t* test). (**D**) Representative images from the quantification are shown. U87 cells were infected with Lv-NC or Lv-miR-129 for 72 hours and then transfected with pDsRed-LC3 for another 24 hours, or treated with 100 nM Rap and transfected with pDsRed-LC3 for 24 hours. Scale bars represent 20 μm. Quantification of the cells with red fluorescent dots (mean ± SEM of independent experiments, *n* = 4, ***P < 0.001, Student's 2-tailed *t* test). (**E**) Transmission electron microscope representative images of U87 cells infected with Lv-NC or Lv-miR-129 for 96 hours or treated with 100 nM Rap for 24 hours. Top panel: images (×5000 magnification). Scale bars represent 2 μm. Bottom panel: close-up images (×10000 magnification). Scale bars represent 1 μm. (**F**) Treatment with or without 3-MA for 24 hours after infected with Lv-NC or Lv-miR-129 for 72 hours in U87 cells. The LC3-II/LC3-I ratios from immunoblots were measured by Image J densitometric analysis (mean ± SEM of independent experiments, *n* = 3, *P < 0.05).

Many studies have shown that miR-129 acted as a tumor suppressor in a variety of human cancers [15–17]. It has also been reported that miR-129 was decreased in clinical glioma samples by miRNA microarray and had an antiproliferative effect in glioma cell lines using a human pre-miR miRNA precursor library [26]. To investigate the influence of miR-129 on autophagy, we infected with pHAGE-miR-129 lentivirus (Lv-miR-129) in U87 cells (named U87-129) and U251 cells (named U251-129) cells, which stably expressed miR-129. We also infected with pHAGE lentivirus (Lv-NC) in U87 cells (named U87-NC) and U251 cells (named U251-NC) cells as negative controls, respectively. After infected with Lv-NC or Lv-miR-129 lentivirus for 96 hours, the infection efficiency was nearly 96% as indicated by fluorescence microscope scan and flow cytometry (Supplementary Figure S1A and S1B), and the expression of miR-129 were present at a high level in U87-129 and U251-129 cells (Supplementary Figure S1C). Then, we detected the expression of LC3-I (microtubule-associated protein 1 light chain 3) and its membrane-bound form, LC3-II, by Western blot and found that the conversion of LC3-I to LC3-II increased in both U87-129 and U251-129 cells compared with control cells (Figure 1A). Moreover, miR-129 promoted LC3 conversion in a time dependent manner. The conversion of LC3-I to LC3-II increased after infected with Lv-miR-129 for 48, 72 and 96 hours in U87 cells (Supplementary Figure S2A). On the contrary, U87 or U251 cells were transfected with miR-129 sponge vector, which inhibits endogenous miR-129, or scramble sponge vector for 48 hours. Then, cells were treated with rapamycin (Rap) for an additional 24 hours. The miR-129 sponge (129 sponge) vector effectively inhibited the expression of miR-129 (Supplementary Figure S1D) and Rap-induced the amount of LC3-I converted to LC3-II compared with scramble sponge (SCR sponge) vector (Figure 1B).

Next, we examined the levels of p62, a poly-ubiquitin binding protein that binds to LC3 and is degraded by autophagy to determine whether miR-129-induced autophagosome accumulation is due to autophagy induction or a block in downstream steps. As shown in Figure 1A, nearly 40% of p62 was degraded in both U87-129 and U251-129 cells compared with control cells, suggesting that miR-129 promotes autophagic degradation. Moreover, miR-129 promoted p62 degradation in a time dependent manner. The p62 was degraded after infected with Lv-miR-129 for 48, 72 and 96 hours in U87 cells (Supplementary Figure S2A). Knockdown of endogenous miR-129 expression prevented p62 degradation by Rap (Figure 1B). Furthermore, cells were treated with the lysosomotropic reagent chloroquine (CQ) to block autophagic degradation. CQ treatment caused significant increase of LC3-II in both Lv-NC and Lv-miR-129 infected cells (Supplementary Figure S2B). The protein levels of p62 in Lv-miR-129 infected cells were also upregulated by CQ (Supplementary Figure S2B). Thus, these results demonstrate that enforced expression of miR-129 increases autophagic flux.

Our research also suggests that the mRNA levels of some autophagy-related genes (Atgs) such as Atg5, Atg7, Atg12, Atg14 and BECN1 (also known as Atg6) increased from 2 to 2.5 in U87-129 cells compared with U87-NC cells according to quantitative PCR (qPCR) results (Figure 1C). In addition, we transfected with pDsRed-LC3 into U87-NC and U87-129 cells for 24 hours, meanwhile U87-NC cells were treated with 100 nM Rap for 24 hours as a positive control. We then analyzed the samples by confocal microscopy. The results showed an accumulation of pDsRed-LC3 puncta in the cytoplasm of U87-129 cells and Rap-treated U87-NC cells but not that of untreated U87-NC cells (Figure 1D). Besides, transmission electron microscope results indicated that many double-layered membrane autophagosomes accumulated in the cytoplasm of U87-129 cells and Rap-treated U87-NC cells (Figure 1E). Treatment with 3-Methyladenine (3-MA), an autophagy inhibitor due to inhibit PI3K, in U87-129 cells could restore miR-129-induced autophagic flux (Figure 1F). Therefore, these data demonstrated that overexpression of miR-129 increased the autophagic activity of glioma cells.

### MiR-129 induced autophagic flux by targetedly suppressing Notch-1

**Figure 2:**
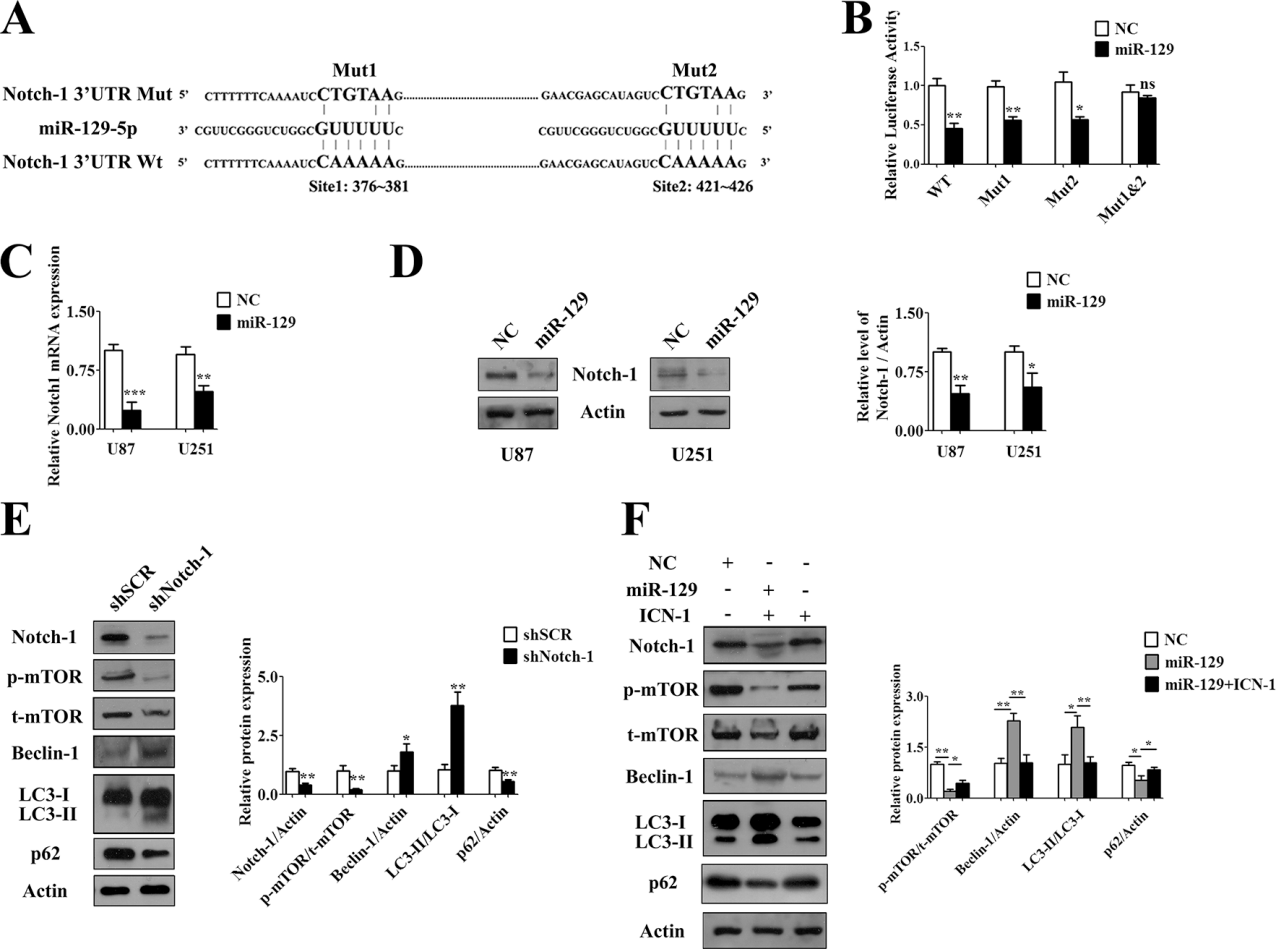
MiR-129 induced autophagy by targetedly suppressing Notch-1 (**A**) Predicted binding sequences between miR-129 and seed site in Notch-1 3′UTR. (**B**) Normalized luciferase activity in lysates from U87 cells cotransfected with wild-type (WT) or mutant Notch-1 luciferase constructs (Mut1, Mut2 and Mut1-2) and scramble mimics (NC) or hsa-miR-129-5p mimics (miR-129) (mean ± SEM of independent experiments, *n* = 3, *P < 0.05, **P < 0.01, ns, not significant). (**C**) Results of qPCR analysis of Notch-1 mRNA level after Lv-NC or Lv-miR-129 infection of U87 or U251 cells for 72 hours. (mean ± SEM of independent experiments, *n* = 3, **P < 0.01, ***P < 0.001). The results were expressed as fold change relative to GAPDH mRNA levels. (**D**) Notch-1 protein levels were decreased after U87 or U251 cells were infected with Lv-miR-129 for 96 hours. Notch-1/Actin ratios were calculated using image J densitometric analysis (mean ± SEM of independent experiments, *n* = 3, *P < 0.05, **P < 0.01). (**E**) Transfection with shSCR or shNotch-1 for 72 hours in U87 cells. Western blot analysis of Notch-1, phosphorylated mTOR (p-mTOR), total mTOR (t-mTOR), Beclin-1, LC3 and p62 degradation (mean ± SEM of independent experiments, *n* = 3, *P < 0.05, **P < 0.01). (**F**) The U87 cells were infected with Lv-NC or Lv-miR-129 for 48 hours, and then transfected with exogenous ICN-1 for another 48 hours. The protein levels of Notch-1, p-mTOR, t-mTOR, Beclin-1, p62 and LC3 were analyzed by Western blot (mean ± SEM of independent experiments, *n* = 3, *P < 0.05, **P < 0.01).

Using DIANA microT v3.0 and miRanda bioinformatics tools, we found that hsa-miR-129-5p but not hsa-miR-129-3p potentially targets Notch-1, which contains two “seed” regions in the 3′UTR (Figure 2A). Then, we designed pMIR-reporter constructs containing either wild-type (WT) or mutated-type (Mut) hsa-miR-129-5p binding sites at the 3′UTR of Notch-1. The luciferase reporter assay was performed 24 hours after transfection. Hsa-miR-129-5p mimics the significantly reduced luciferase activity of WT, mutated-type 1 (Mut1), or mutated-type 2 (Mut2) Notch-1 3′UTR compared with scramble mimics but had no significant effect on mutated-type 3 (mutate both binding sites) (Figure 2B). Moreover, the expression of Notch-1 mRNA (Figure 2C) and protein (Figure 2D) were both obviously downregulated in U87-129 and U251-129 cells compared with negative controls. On the contrary, miR-129 inhibition upregulates the protein level of Notch-1 in glioma cells (Supplementary Figure S3A and S3B).

A recent report showed that inhibition of mTOR-STAT3-Notch-1 signaling induced autophagic cell death in glioma cells [22]. To further determine whether Beclin-1 was involved in Notch-1 inhibition-induced autophagy, we performed a Western blot assay and verified that autophagy was induced after knockdown of Notch-1 along with decreased mTOR phosphorylation and Beclin-1 overexpression (Figure 2E). Then, we performed rescue experiments by overexpressing the intracellular domain of Notch-1 (ICN-1). The result showed that miR-129-induced autophagy and related protein expression was rescued to control levels after cotransfection of ICN-1 (Figure 2F). Taken together, these results demonstrated that inhibition of endogenous Notch-1 might promote miR-129-induced autophagy through suppress the activity of mTOR and enhance the expression of Beclin-1.

### E2F7 partially promoted Notch-1 inhibition-induced autophagy by upregulating Beclin-1

**Figure 3:**
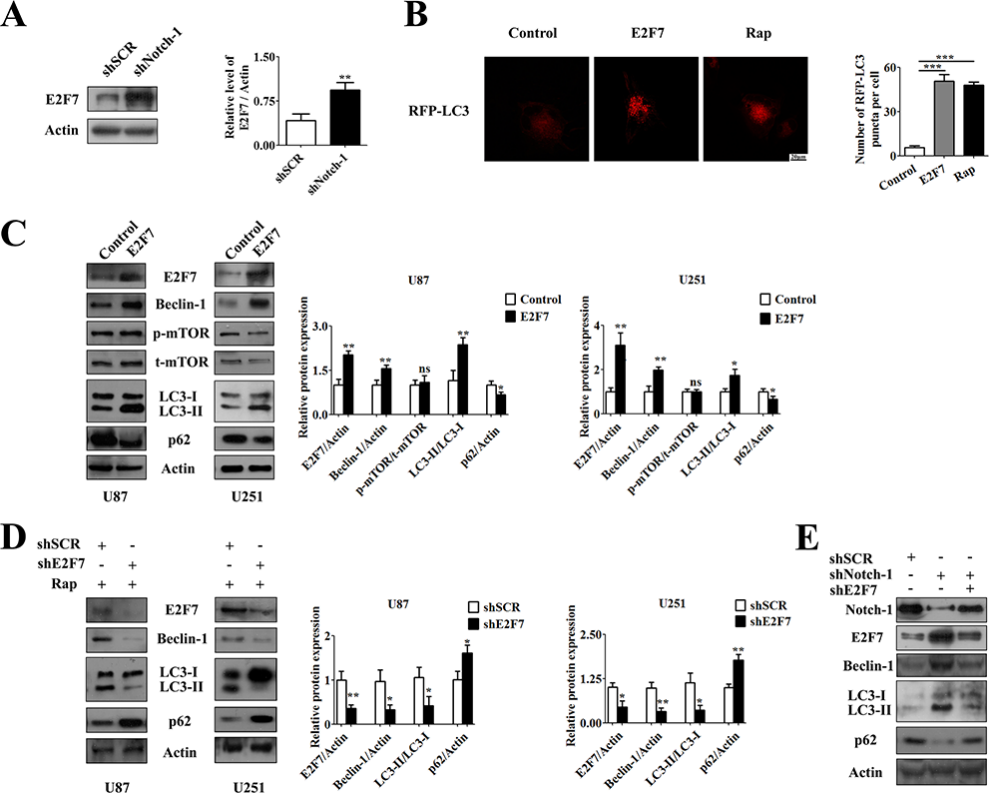
E2F7 was suppressed by Notch-1 and induced autophagy (**A**) Knockdown of Notch-1 promotes the expression of E2F7. U87 cells were transfected with indicated shRNA for 72 hours. The expression of E2F7 was analyzed by Western blot. E2F7 expression from immunoblots were measured by Image J densitometric analysis (mean ± SEM of independent experiments, *n* = 3, **P < 0.01). (**B**) Representative images from the quantification were shown. U87 cells were transfected with E2F7 overexpression vector or pcDNA3.1 control vector for 48 hours and then transfected with pDsRed-LC3 for 24 hours, or treated with 100 nM Rap for 24 hours. Scale bars represent 20 μm. Quantification of the cells is shown with red fluorescent dots (mean ± SEM of independent experiments, *n* = 4, **P < 0.01, Student's 2-tailed *t* test). (**C**) Transfection of E2F7 overexpression vector or pcDNA3.1 control vector into U87 and U251 cells for 48 hours. The expression of E2F7, Beclin-1, p-mTOR, t-mTOR, p62 and LC3 was tested by Western blot, then was measured by Image J densitometric analysis of their protein expression from immunoblots (mean ± SEM of independent experiments, *n* = 3, *P < 0.05, **P < 0.01). (**D**) Transfection with shNC or shE2F7 vector into U87 or U251 cells for 48 hours, then treatment with 100 nM Rap for another 24 hours. The expression of Beclin-1, p62 and LC3 was tested by Western blot (mean ± SEM of independent experiments, *n* = 3, *P < 0.05, **P < 0.01). (**E**) U87 cells were transfected with indicated shRNA for 72 hours. Beclin-1, LC3 and p62 were identified by immunoblot analysis.

Having tested the influence of Notch-1 on autophagy as indicated by Ingenuity Pathway Analysis (IPA) in Gastric cancer network 1: http://www.ncbi.nlm.nih.gov/biosystems/760635, Western blot analysis was conducted to detect the impact of Notch-1 on E2F7 expression. The result demonstrated that knockdown of endogenous Notch-1 upregulated E2F7 (Figure 3A). Next, we investigated the influence of E2F7 on autophagy. The efficiency of E2F7 relative expression in E2F7 vector-transfected cells was very high (Supplementary Figure S4A), whereas the E2F7-interfering vector presented the highest inhibition efficiency tested by qPCR (Supplementary Figure S4B). Confocal microscopy results showed a pDsRed-LC3 puncta accumulation in E2F7-overexpressed and Rap-treated cells but not control cells (Figure 3B). Moreover, Western blot showed that overexpression of E2F7 promoted the conversion of LC3-I to LC3-II, p62 degradation and Beclin-1 upregulation, but had no obvious effect on mTOR activity (Figure 3C). Furthermore, CQ treatment caused significant increase of LC3-II in both pcDNA3.1 and E2F7 vector transfected cells (Supplementary Figure S4D). In addition, the protein levels of p62 in E2F7 transfected cells were also upregulated by CQ (Supplementary Figure S4D). Instead, knockdown of endogenous E2F7 suppressed the conversion of LC3-I to LC3-II, p62 degradation and Beclin-1 expression during Rap treatment, indicating a decrease of autophagic activity (Figure 3D).

Next, we aim to clarify whether the function of Notch-1 in autophagy was related to E2F7. The results showed that knockdown of endogenous E2F7 rescued the protein levels of Beclin-1 and autophagic flux triggered by shNotch-1 back to control levels in U87 cells (Figure 3E). In light of these results, we demonstrate that E2F7 was a probable inducer of autophagy and partially promoted Notch-1 inhibition-induced autophagy.

### MiR-129 triggered autophagic flux partially via the Notch-1/E2F7/Beclin-1 axis

**Figure 4:**
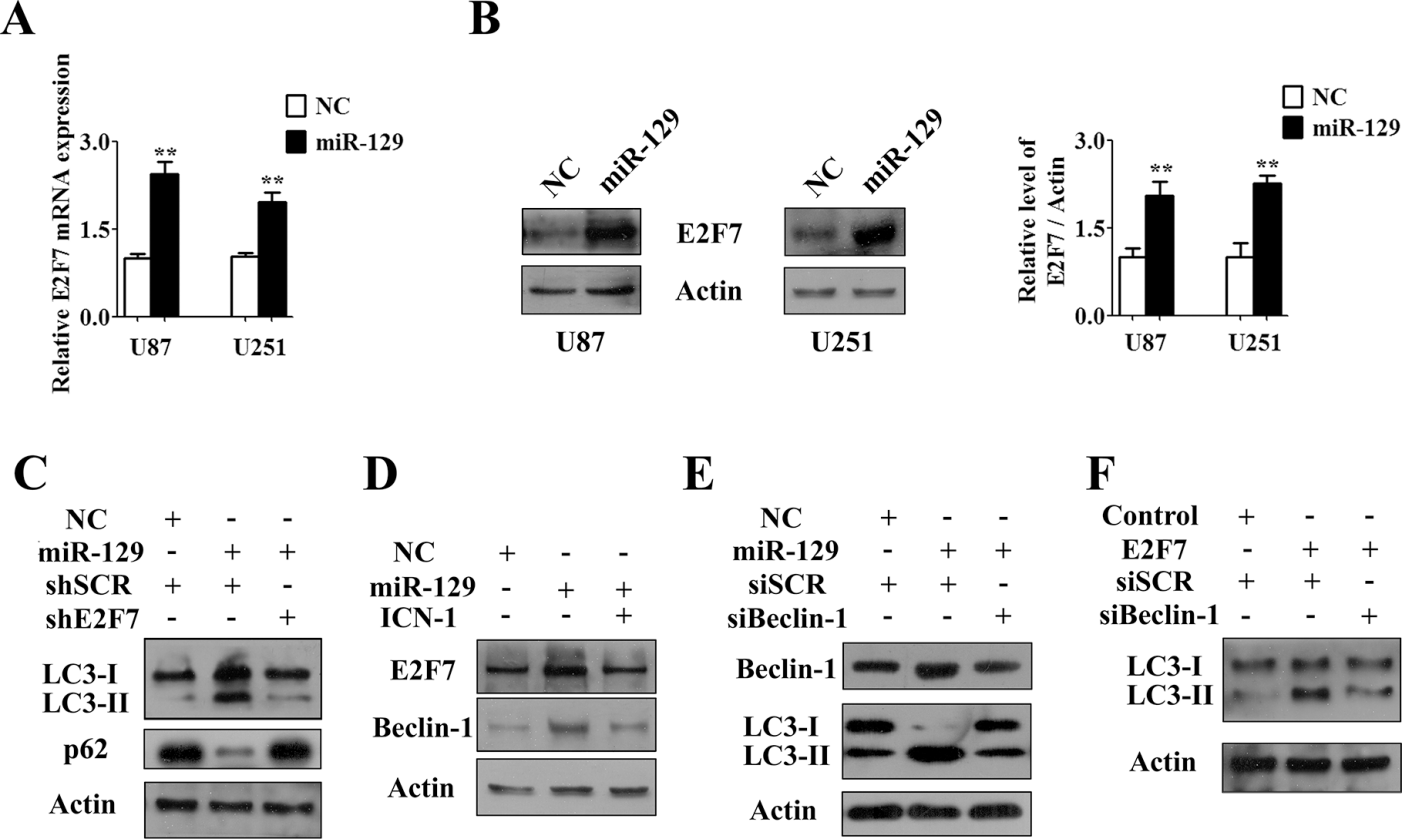
Notch-1/E2F7/Beclin-1 axis was involved in miR-129-triggered autophagic flux (**A**) qPCR analysis of relative expression of E2F7 mRNA in U87 and U251 cells after infected with Lv-NC or Lv-miR-129 for 72 hours (mean ± SEM of independent experiments, *n* = 3, **P < 0.01). (**B**) Western blot analysis of E2F7 protein levels in U87 and U251 cells that were infected with Lv-NC or Lv-miR-129 for 96 hours. E2F7/Actin ratios were calculated using image J densitometric analysis (mean ± SEM of independent experiments, *n* = 3, **P < 0.01). (**C**) U87 cells were infected with Lv-NC or Lv-miR-129 for 24 hours and then transfected with indicated shRNA for another 72 hours. The conversion of LC3-I to LC3-II and degradation of p62 were analyzed by Western blot. (**D**) U87 cells were infected with Lv-NC or Lv-miR-129 for 48 hours and then enforced expression of ICN-1 or not for another 48 hours. E2F7 and Beclin-1 levels were detected by Immunoblot analysis. (**E**) U87 cells were infected with Lv-NC or Lv-miR-129 for 24 hours and then transfected with indicated siRNA (50 nM) for another 72 hours. The expression of Beclin-1 and the conversion of LC3-I to LC3-II were analyzed by Western blot. (**F**) U87 cells were cotransfected with E2F7 overexpression vector or pcDNA3.1 control vector and/or indicated siRNA (50 nM) for 72 hours. The conversion of LC3-I to LC3-II was analyzed by Western blot.

Consistent with the results shown above, miR-129 could promote the mRNA and protein expression of E2F7 both in U87 and U251 cell lines (Figure 4A and 4B). However, a qPCR analysis of U87 cells revealed that overexpression of E2F7 had no obvious impact on the expression of miR-129 compared with negative controls (Supplementary Figure S4C). Moreover, transfection of shE2F7 into U87-129 cells suppressed LC3-I conversion to LC3-II and rescued p62 degradation to control levels compared with U87-129 transfected with shNC (Figure 4C). These results indicated that E2F7 partially promoted miR-129-triggered autophagic flux.

In addition, forced expression of ICN-1 restored E2F7 and Beclin-1 protein levels induced by miR-129 (Figure 4D). Knockdown of Beclin-1 rescued E2F7- and miR-129-induced autophagic flux (Figure 4E and 4F). Therefore, these results suggested that miR-129 likely induced autophagic flux partially in a Notch-1/E2F7/Beclin-1-dependent manner in U87 cells.

### Inhibition of miR-129-induced autophagic flux rescued the viability of glioma cells

**Figure 5:**
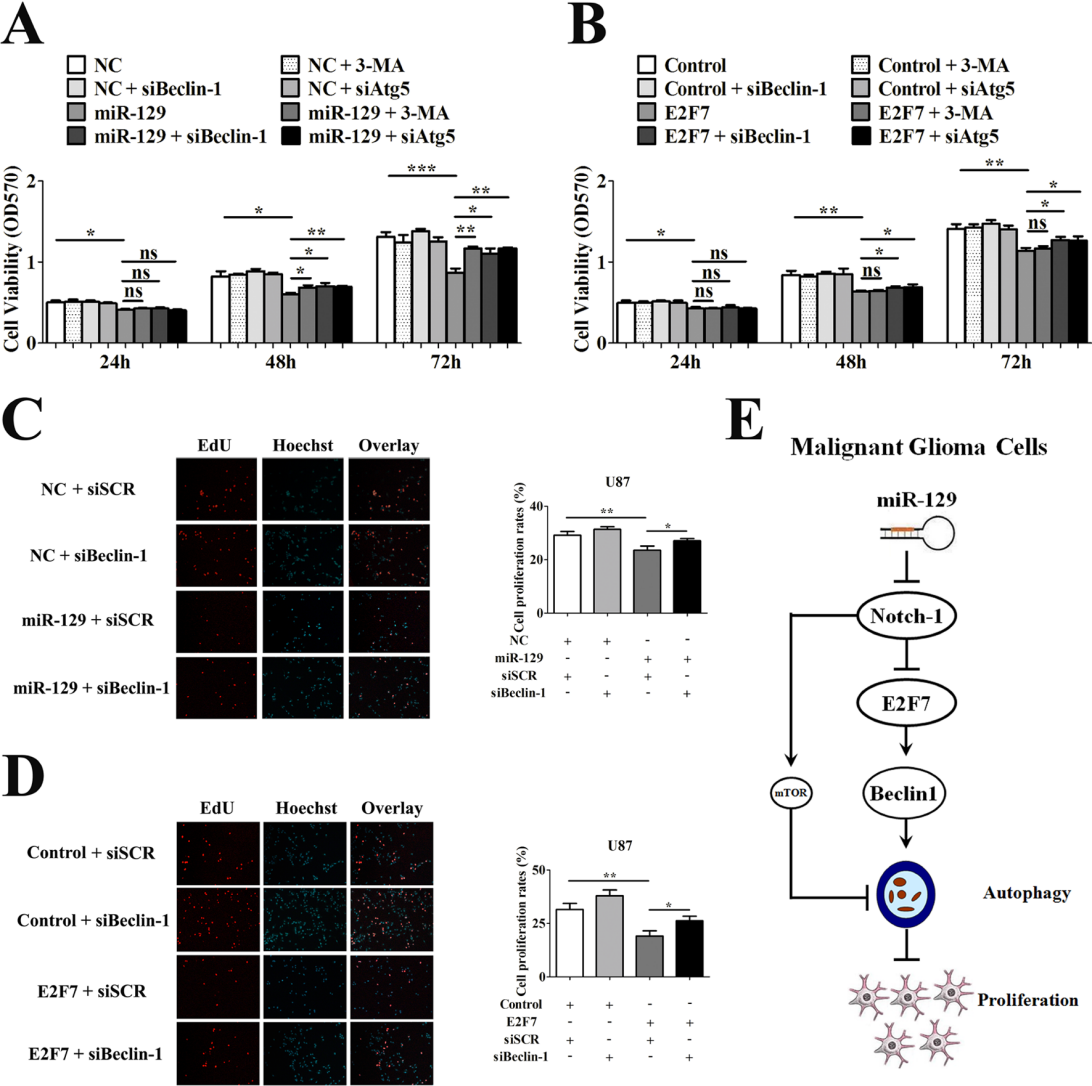
Suppression of miR-129-induced autophagic flux rescued cell viability of U87 cells (**A**) U87 cells were infected with Lv-NC or Lv-miR-129 for 24 hours and then treated with 5 mM 3-MA or transfected with 50 nM siBeclin-1 or siAtg5 for another 24 hours, 48 hours and 72 hours. The cell viability was tested by MTT assay (mean ± SEM of independent experiments, *n* = 4, *P < 0.05, **P < 0.01, ***P < 0.001, ns, not significant Student's 2-tailed *t* test). (**B**) Cotransfection with 3 μg indicated vectors or 50 nM siRNA and then treated with 5 mM 3-MA or transfected with 50 nM siBeclin-1 or siAtg5 for another 24 hours, 48 hours and 72 hours in U87 cells. The cell viability was tested by MTT assay (mean ± SEM of independent experiments, *n* = 4, *P < 0.05, **P < 0.01, ns, not significant, Student's 2-tailed *t* test). (**C**) After infected with Lv-NC or Lv-miR-129 for 24 hours, the U87 cells were transfected with 50 nM siSCR or siBeclin-1 for another 72 hours. The proliferation of U87 cells were tested by EdU assay (mean ± SEM of independent experiments, *n* = 3, *P < 0.05, **P < 0.01). (**D**) After cotransfected with 3 μg control or E2F7 vector and 50 nM siSCR or siBeclin-1 for 24 hours, the U87 cells were transfected with 50 nM siSCR or siBeclin-1 for another 72 hours. The proliferation of U87 cells were tested by EdU assay (mean ± SEM of independent experiments, *n* = 3, *P < 0.05, **P < 0.01). (**E**) Principle signaling pathways involved in miR-129- and E2F7-induced autophagy and cell proliferation inhibition. By targetedly suppressing Notch-1, which in turn suppressed the activation of mTOR and promoted the expression of Beclin-1, miR-129 induced autophagy. E2F7 partially mediated miR-129- and Notch-1 inhibition-induced autophagy. By upregulating Beclin-1 expression, miR-129 and E2F7 induced autophagy and inhibited cell proliferation.

To study the role of miR-129- and E2F7-induced autophagy on viability of glioma cells, we performed MTT assay by using 3-MA, Beclin-1 siRNA (sibeclin-1) or Atg5 siRNA (siAtg5) to block autophagic flux, respectively. The inhibition efficiency of Atg5 siRNA in U87 cells was tested by Western blot (Supplementary Figure S6A and S6B). The MTT results indicated that suppression of miR-129-induced autophagic flux by 3-MA, siBeclin-1 or siAtg5 attenuated the antiproliferative function of miR-129 in U87-129 cells after 48 or 72 hours (Figure 5A). Transfection with siBeclin-1 or siAtg5 also rescued cell viability suppressed by E2F7 in U87 cells (Figure 5B). However, pretreatment with 3-MA had no obvious effect on cell viability suppressed by E2F7, indicating that the mTOR pathway was not responsible for E2F7-induced autophagy (Figure 5B). To further explore the effect of miR-129- and E2F7-induced autophagy on cell proliferation of glioma cells, we performed 5-ethynyl-20-deoxyuridine incorporation (EdU) assay and colony formation assay. Consistent with MTT, the results showed that overexpression of miR-129 or E2F7 could inhibit the proliferation of U87 cells (Figure 5C and 5D, Supplementary Figure S7A and S7B). Suppressed miR-129- or E2F7-induced autophagy by siBeclin-1 could rescue the cell proliferation (Figure 5C and 5D, Supplementary Figure S7A and S7B). These results indicated that miR-129- or E2F7-induced autophagy was injurious to glioma cells.

### MiR-129 treatment suppressed glioma cell growth and induced autophagy in xenograft model

**Figure 6:**
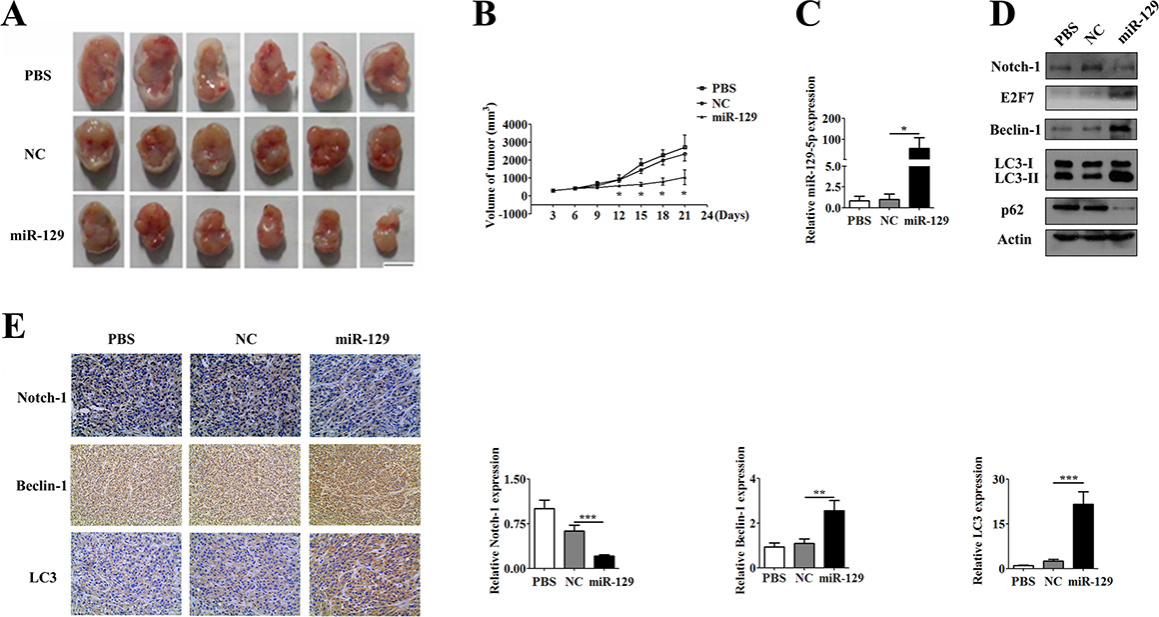
U87 GBM cell xenograft tumor experiment (**A**) The mice were monitored every 3 days for 3 weeks and a comparison of the sizes of the xenograft tumors were found in the different treatment groups. (**B**) The xenograft tumor growth curve showed an anti-tumor effect in miR-129 *in vivo*. Significant reduced volumes of tumor size were observed in miR-129 group compared with PBS or NC group from the 12th day after injected with lentiviruses. The volume of tumor size between PBS and NC groups had no significant difference all the time. (**C**) qPCR analysis of expression of miR-129 in each group of tissues (*P < 0.05). (**D**) Xenograft tumor tissues were tested to detect the expression of Notch-1, E2F7, Beclin-1, LC3 and p62 in PBS group or Lv-NC group or Lv-miR-129 group by Western blotting. (**E**) The expression of Notch-1, Beclin-1 and LC3 in each group was shown by the immunohistochemical staining of xenograft tumors. Protein expression levels of Notch-1, Beclin-1 and LC3 were evaluated by Image Pro Plus 6.0 (IPP6.0) image analysis. (mean ± SEM of 6 different horizons, **P < 0.01, ***P < 0.001).

The *in vitro* experiments demonstrated that miR-129 could enhance autophagic flux in glioma cells. To further confirm this *in vivo*, we performed a xenograft experiment with U87 cells to manifest the inductive effect of miR-129 on autophagy in nude mice. U87 cells were injected subcutaneously into 18 nude mice. Five days later, each nude mouse formed a palpable tumor and they were divided into three groups randomly, with 6 in each group. Then, the groups were injected with phosphate buffered saline (PBS), Lv-NC and Lv-miR-129, respectively. The mice were monitored every 3 days for 3 weeks, and three groups exhibited significant differences by the 8th day after treatment. The average tumor volume was significantly decreased in the Lv-miR-129 treatment group compared with the other two groups (Figure 6A and 6B).

To investigate the role of miR-129, as well as Notch-1, E2F7, Beclin-1, p62 and LC-3 expression in autophagy *in vivo*, tumor tissues were subjected to miR-129 and protein expression analyses. Western blot analysis showed that the Lv-miR-129 group had a high level of miR-129 with downregulated Notch-1 expression along with increased E2F7 and Beclin-1 expression, p62 degradation and LC3-I to LC3-II conversion compared with the other two groups (Figure 6C and 6D). Immunohistochemical analysis showed that Notch-1 expression was downregulated, whereas Beclin-1 and LC3 expression were increased in the Lv-miR-129 group compared with the other two groups (Figure 6E). These data indicate an inducing role for miR-129 in autophagy and the regulation of Notch-1, E2F7 and Beclin-1 by miR-129 *in vivo* and *vitro*.

### MiR-129 negatively correlated with Notch-1 in glioma tissues and cell lines

**Figure 7:**
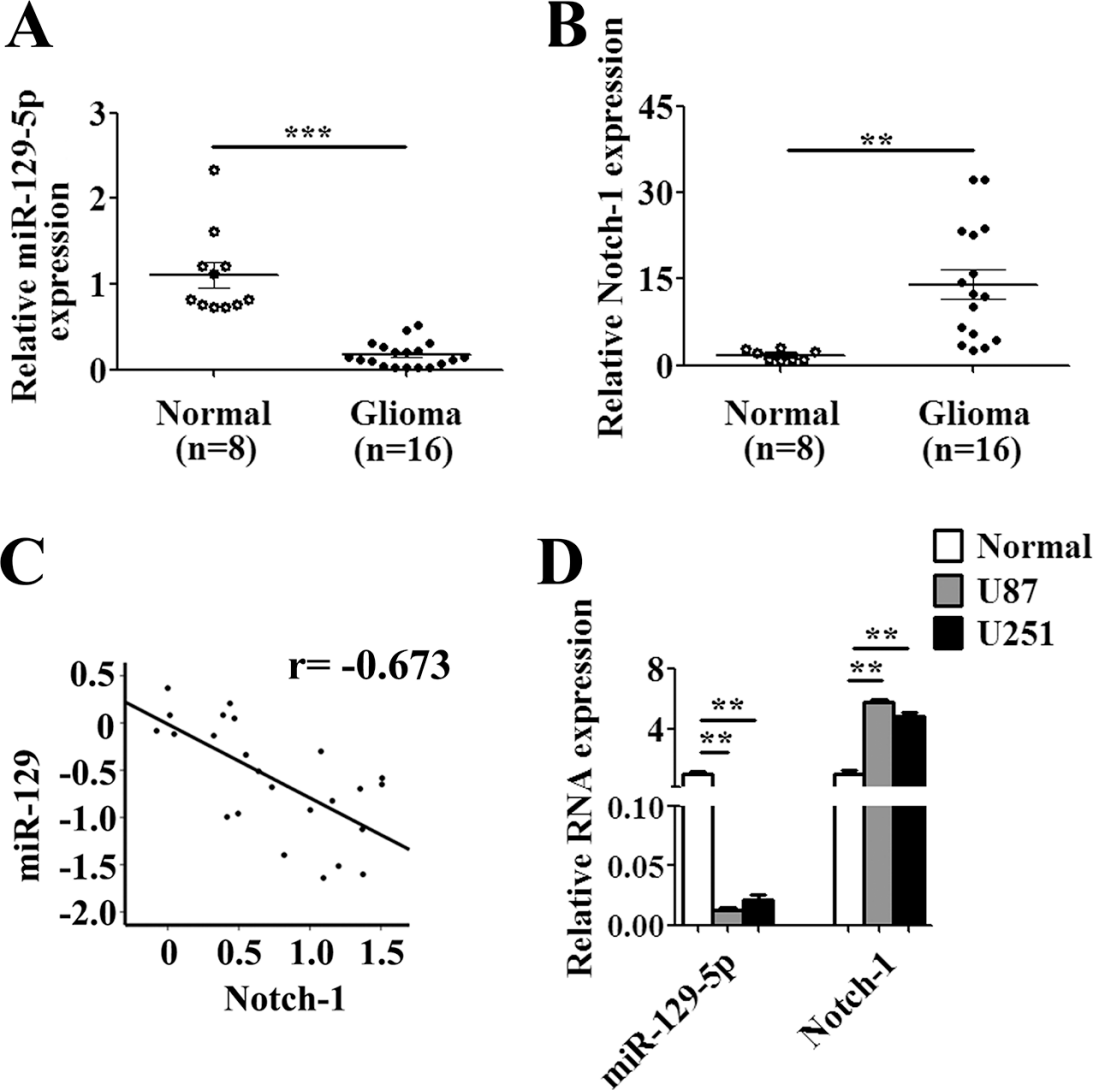
MiR-129 negative correlation with Notch-1 in glioma tissues and cell lines (**A**) Expression of miR-129 and Notch-1 mRNA in 16 malignant glioma and 8 normal brains tissues was analyzed by qPCR, indicating that miR-129 expression was significantly lower and (**B**) Notch-1 was significantly higher in malignant glioma than that of normal brain tissues (**P < 0.01, ***P < 0.001). (**C**) MiR-129 negative correlation with Notch-1 mRNA in glioma tissues (*r* = −0.673). (**D**) qPCR analysis of expression of miR-129 and Notch-1 mRNA in two glioma cell lines compared with normal brain tissues (**P < 0.01).

To further investigate the correlation between miR-129 and Notch-1, the mRNA levels of miR-129 and Notch-1 were detected from 16 clinical high-grade glioma samples (WHO grade III or IV) and two glioma cell lines (U87, U251) compared with 8 normal brain tissues from brain trauma patients. These results showed that miR-129 was expressed in glioma samples and cell lines at a relatively low level, whereas the Notch-1 relative expression was high (Figure 7A, 7C–7D). There is a negative correlation between miR-129 and Notch-1 (Figure 7B). Thus, miR-129 is a promising diagnostic marker in glioma.

## DISCUSSION

Many studies demonstrated that miR-129 acted as a tumor suppressor with the ability to inhibit proliferation and promote apoptosis in a variety of tumor cell lines [15–17]. In this study, the major novel findings were that miR-129 is a new inducer of autophagy both through mTOR signaling and upregulation of Beclin-1 by targetedly suppressing Notch-1 in glioma cell lines. MiR-129 triggered autophagy partially by a novel Notch-1/E2F7/Beclin-1 axis. Suppression of miR-129-induced autophagic flux, which may contributes to its anti-tumor effect, increased the viability and proliferation of glioma cells.

There were two potential “seed” sites in the 3′UTR of Notch-1 for hsa-miR-129-5p (Figure 2A), but there were no possible “seed” sites for hsa-miR-129-1-3p. As miR-129-1 was frequently deleted in solid tumors [27], it was cloned into a pHAGE-CMV-IZsGreen vector (pHAGE) pHAGE-miR-129 and named as pHAGE-miR-129 in our experiments. It has been demonstrated that miR-129 acted as a tumor suppressor to inhibit proliferation and promote apoptosis in a variety of tumor cell line [15–17]. However, whether miR-129 affected autophagy was unclear. To our surprise, the autophagic flux was obviously higher in both U87-129 and U251-129 cells than their control cells. To further confirm this result, the autophagic flux of U87-129 cells was tested at different time points and the results showed an increased LC3 conversion and p62 degradation at 48, 72 and 96 hours (Supplementary Figure S2A). These results showed that the autophagic flux induced by miR-129 was a continual process.

Notch-1 is critical for the development of normal brain and glioma tissues [18, 19]. Inhibition of Notch-1 led to delayed tumor growth and longer survival [28]; it not only protected cardiomyocytes from ethanol-induced autophagy [29] but also mediated oroxylin A-induced autophagic cell death in glioma cells [22]. In line with previous studies, we confirmed that knockdown of Notch-1 induced autophagy in glioma cells. It has been demonstrated that Notch-1 promoted proliferation and survival of glioma cells through EGFR/Mcl-1 and mTOR signaling [20, 21]. It indicated that suppress the expression of Notch-1 may induce autophagy in glioma. However, whether Notch-1 inhibition could influence Beclin-1 expression was unclear. Indeed, we demonstrated that overexpression of miR-129 or knockdown of Notch-1 not only inhibited mTOR activity but also increased Beclin-1 protein levels in glioma cells. Thus, Beclin-1 may be involved in miR-129 or Notch-1 inhibition-induced autophagy (Figure 5E).

E2F family members were involved in a wide range of biological activities, including cell cycle progression, differentiation and apoptosis [30]. To date, only E2F1 had been linked to the regulation of autophagy [24, 25, 31, 32]. Moreover, Li et al. suggested that there is a feedback loop involving E2F7 and E2F1 [33]. However, the role of E2F7 in autophagy is unclear. In the present study, we first showed that E2F7 could trigger autophagic flux in glioma. Consistent with the result of miR-129 overexpression and Notch-1 inhibition, E2F7 also increased Beclin-1 protein levels and induced autophagy in U87 and U251 glioma cells. Surprisingly, overexpression of E2F7 had no obvious effect on the expression and activity of mTOR. Therefore, the effect of E2F7 on autophagy might be context dependent.

The Ingenuity Pathway Analysis (IPA) indicated a possible regulation of E2F7 by Notch-1 via NF-κB in Gastric cancer network 1. Thus, we tested the effect of Notch-1 on E2F7, and Western blot showed that knockdown of Notch-1 upregulated E2F7 expression. Interestingly, knockdown of endogenous E2F7 upregulated Notch-1 protein level (date not shown). There might have a feedback loop between Notch-1 and E2F7. It may be relevant to the Notch-1 expression could be upregulated at transcriptional level by E2F1 in hepatocellular carcinoma cells [34] and the feedback loop involving E2F7 and E2F1 [33]. In addition to Notch-1 and E2F7, we also found miR-129 could inhibit the expression of Notch-2, E2F1 and E2F3 while promoted the expression of E2F8 (Supplementary Figure S5A and S5B). It is unclear, however, whether miR-129 induced autophagy partially by suppressing of Notch-2 or there have a feedback loop between Notch-2 and E2F7 need to be investigated.

Many studies have demonstrated the antiproliferative effect of miR-129 in a variety of human cancers [15–17]. Moreover, a small number of studies had reported an antiproliferative role of E2F7 through repression of E2F1 [35–37] or interaction with CtBP [38]. High E2F7 expression was shown to improve clinical outcomes and was a favorable disease-free survival factor in ovarian cancer, indicating that E2F7 improve the prognosis of ovarian cancer [39]. However, the influence of miR-129- or E2F7-induced autophagy on cell proliferation was unclear. In our study, the MTT results showed that inhibition of autophagic flux by treatment with 3-MA rescued the viability of U87-129 cells compared with control cells. However, no difference in activity recovery was observed between E2F7-transfected cells treated with 3-MA and untreated cells. These results indicated that Beclin-1 rather than mTOR signaling may contributes to the antiproliferative effect of E2F7. Previous research has shown decreased expression of Beclin-1 in high-grade gliomas [40]. More importantly, Beclin-1 played a role in autophagy and functioned as a tumor suppressor in malignant glioma cells [41, 42]. In our study, transfection of siBeclin-1 significantly rescued the proliferation of U87-129 cells and E2F7 transfected U87 cells compared with their control cells. These results suggested that miR-129 or E2F7 had an antiproliferative function may partially by inducing Beclin-1-mediated autophagy (Figure 5E).

In summary, our findings document miR-129 and E2F7 as two autophagy inducers. Importantly, inhibition of autophagic flux induced by miR-129 or E2F7 rescued the proliferation of glioma cells. Further research is needed to explore whether this mechanism is consistently observed in other types of cells. These findings suggest that miR-129 is a promising therapeutic target as well as a diagnostic marker in glioma.

## CONCLUSION

In conclusion, the present study demonstrated that miR-129 and E2F7 as new inducer of autophagy. MiR-129 induced autophagy through mTOR signaling by targetedly suppressing Notch-1 in glioma cell lines. Moreover, miR-129 triggered autophagy partially by a novel Notch-1/E2F7/Beclin-1 axis. Suppressed miR-129-induced autophagic flux increased the viability of glioma cells which may further illustrate the mechanism of miR-129 in contributing to cell proliferation. These findings may provide new insights to the application of miR-129 for glioma therapy.

## MATERIALS AND METHODS

### Human tissue samples

All tissues were collected from the Department of Neurosurgery, Renmin Hospital of Wuhan University (Wuhan, China) for RT-PCR analysis. Sixteen primary GBM tissues were obtained from the surgery and immediately stored in liquid nitrogen until use. Patients did not receive radiotherapy and chemotherapy before surgery. Eight normal brain tissues were obtained from patients with cerebral trauma. The study was approved by the ethics committee of Renmin Hospital.

### Cell line culture

293T (human embryonic kidney cells), U87 and U251 cells (Human GBM cells) were purchased from American Type Culture Collection (ATCC, Manassas, VA, USA). Cell lines were cultured in Dulbecco's modified Eagle's medium (Invitrogen, San Diego, CA, USA) containing 10% fetal bovine serum (Invitrogen, San Diego, CA, USA) in a 5% CO_2_ humidified incubator at 37°C.

### Target prediction

Target genes of hsa-miR-129-5p were predicted using multiple target prediction algorithms: http://diana.cslab.ece.ntua.gr/microT/ and http://www.microrna.org/microrna/home.do. The predicted targets were further demonstrated by a variety of biological experiment.

### miRNA, siRNA, shRNA, and transfections

Cells were transfected with miR-129 mimics or non-specific mimics as a negative control (NC) (RiboBio, Guangzhou, China) using Lipofectamine^™^ 2000 reagent (Invitrogen, San Diego, CA, USA) according to the manufacturer's protocol. DNA sequences targeting Notch-1 or E2F7 were cloned into pLKO.1. Mature antisense sequences were: Notch-1: 5′- CAAAGACATGACCAGTGGCTA -3′, E2F7: 5′- CTGGACCTGATAGATTATAAA -3′, scramble: 5′- CAACAAGATGAAGAGCACCAA -3′. The sequence of siAtg5 was CAACUUGUUUCACGCUAUAdTdT. Scramble siRNA (siSCR) and siBeclin-1 were purchased from RiboBio.

### Luciferase assay

The 600-bp fragment of the predicted miR-129-binding sequence or a mismatch sequence in the 3′UTR of Notch-1 mRNA, amplified from 293T genomic DNA, was cloned into *Spe* I and *Hand* III restriction site of pMIR-REPORT plasmid (Invitrogen, San Diego, CA, USA). The predicted target site was mutated by site-directed mutagenesis, and 50 nM miR-129 mimics or NC mimics (RiboBio, Guangzhou, China) was transfected into cells with 5 ng Renilla plasmid (Promega, Madison, Wisconsin, USA) and 100 ng of the WT or MUT plasmid. Twenty-four hours before transfection, 6 × 10^4^ cells were plated in 24-well plate. A luciferase assay was performed 24 hours after transfection using the dual-luciferase reporter assay system (Promega, Madison, Wisconsin, USA). The firefly luciferase activity was normalized to Renilla luciferase activity.

### RNA quantification

Total RNA, including miRNAs, was extracted from human tissues, mouse xenografts and cell lines using the TRIzol reagent (Invitrogen, San Diego, CA, USA) according to the manufacturer's protocol. RNAs were reverse-transcribed using the RevertAid First Strand cDNA Synthesis Kit (Thermo, Waltham, MA, USA). All primers are listed in Supplementary Table 1. For quantification of miR-129, the Bulge-LoopTM miRNA qRT-PCR Primer Kits (Ribobio, Guangzhou, China) were utilized following the manufacturer's instructions. qPCR was performed using SuperReal PreMix (SYBR Green) (Tiangen, Beijing, China) with an iCycler thermal cycler (ABI QuantStudio™6 FLEX, USA). The expression of mRNA or miRNA was defined from the threshold cycle (Ct), and relative expression levels were calculated using the 2^−ΔΔCt^ method after normalization with reference to the expression of GAPDH or U6 snRNA.

### Lentivirus production and stable cell lines construction

A fragment of pri-miR-129 was amplified from 293T genomic DNA and cloned into lentiviral vector pHAGE-CMV-MCS-IZsGreen. The empty vector was used as a control. To produce viruses, the pri-miR-129 expression plasmid and the backbone plasmids pMD2.G and psPAX2 were co-transfected into 293T cells using turbofect (Thermo, Waltham, MA, USA). Supernatants containing the viruses were harvested at 48 and 72 hours. U87 and U251 cells were infected with the viruses along with 5 ug/ml polybrene. The transfection efficiency was determined by monitoring GFP expression. The infection efficiency was confirmed by Flow Cytometer (BD AriaIII, USA) examination and qPCR analysis after infected for 72 hours.

### Colony formation assay

For the colony formation assay, the U87 cells were seeded at a density of 500 cells per well in 6-well plates. Twenty-four hours later, the cells were transfected with 50 nM miRNA mimics or siRNAs or 3 μg vectors for 6 hours. Then, the cells were washed with PBS and the cell medium were changed to fresh 10% fetal bovine serum (FBS) and cultured for another 2 weeks. The culture medium was replaced every 3 days. The colony number in each well was calculated.

### 5-ethynyl-20-deoxyuridine incorporation assay

U87 cells were cultured in 96-well plates at 6 × 10^3^ cells per well, infected with lentiviruses for 96 hours or transfected with 50 nM siRNAs or 3 μg vectors for 72 hours. Then, cells were exposed to 50 μM of 5-ethynyl-20-deoxyuridine (EdU, Ribobio, China) for additional 2 hours at 37°C. The cells were fixed with 4% formaldehyde for 30 minutes and treated with 0.5% Triton X-100 for 10 minutes at room temperature. After washing with PBS, the cells of each well were reacted with 100 μL of 1 × Apollo^®^ reaction cocktail for 30 minutes. At last, the DNA contents were stained with 100 μL of 1 × Hoechst 33342 for 30 minutes and observed by fluorescent microscope.

### Confocal microscopy analysis

Cells were transfected with pDsRed-LC3. Twenty-four hours after transfection, cells were fixed and immediately analyzed by confocal microscopy (Lerca-LCS-SP8-STED, Germany). Positive controls were treated with the autophagy inducer, rapamycin, at 100 nM for 24 hours. RFP-LC3 puncta formation, the localization of RFP-LC3 were observed. The number of RFP-LC3 dots per cell was counted.

### Transmission electron microscopy analysis

Cells were fixed in 2% glutaraldehyde in 0.05 M sodium phosphate buffer (pH7.2) for 24 h. After extensive washing in 0.15 M sodium cacodylate buffer (pH7.2) three times, specimens were fixed in 1% OsO_4_ in 0.12 M sodium cacodylate buffer (pH7.2) for 2 hours. The samples were dehydrated in an increasing gradient of ethanol, transferred to propylene oxide, embedded and sliced. Sections were stained with uranyl acetate and lead citrate to observe autophagosomes with a transmission electron microscope (HT7700, Japan). Micrographs were taken at × 5,000 or × 10,000 magnifications.

### Western blotting

Cells and tissues were lysed in RIPA Lysis Buffer (50 mM Tris-Cl pH 7.4 150 mM NaCl 1% Triton X-100 1% sodiumdeoxycholate 0.1% SDS), supplemented with complete protease inhibitor mixture (P0013B, Beyotime Shanghai, China). Then, 20∼30 μg protein/lane was separated on an 8% or 12.5% Tris-Glycine gel and transferred onto a nitrocellulose membrane. The primary antibodies used for Western blotting were as follows: LC3 (Cell Signaling, Beverly, MA; 1:1000), p62 (Cell Signaling, Beverly, MA; 1:2000), Notch-1 (Cell Signaling, Beverly, MA; 1:2000), E2F7 (Abcam, Cambridge, UK; 1:500), Beclin-1 (Cell Signaling, Beverly, MA; 1:1000), mTOR (Cell Signaling, Beverly, MA; 1:1000), p-mTOR (Cell Signaling, Beverly, MA; 1:1000), Atg5 (Cell Signaling, Beverly, MA; 1:1000) and Actin (Abcam, Cambridge, UK; 1:2000).

### Statistical analysis

All experiments were repeated at least three times. The statistical significance between two groups was assessed using Student's 2-tailed *t* test. One-way ANOVA followed by a Bonferroni-Dunn test was used for the comparison of more than two groups. Correlations between the expression levels of miR-129 and Notch-1 were analyzed using Pearson's correlation coefficient. Data were expressed as the mean + standard error of the mean (SEM). *P* < 0.05 was considered significant.

## SUPPLEMENTARY MATERIALS FIGURES AND TABLE


