# Supplementary material for: MiR-129 triggers autophagic flux by regulating a novel Notch-1/ E2F7/Beclin-1 axis to impair the viability of human malignant glioma cells
Source: Oncotarget. 2016 Jan 25;7(8):9222–35. doi: 10.18632/oncotarget.7003 (PMC4891036; doi:10.18632/oncotarget.7003)
Supplement: Supplementary file 1 [file oncotarget-07-9222-s001.pdf]

MiR-129 triggers autophagic flux by regulating a novel Notch-1/E2F7/Beclin-1 axis to impair the viability of human malignant glioma cells

Supplementary Materials

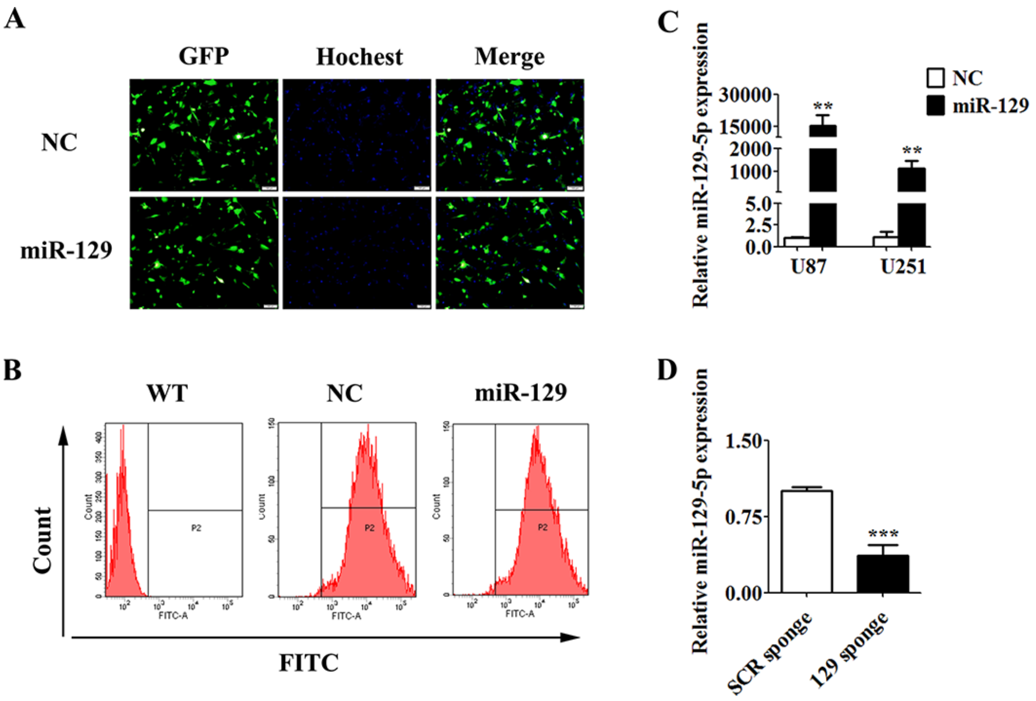

Supplementary Figure S1: Overexpression or inhibition efficiency of miR-129 in human glioma cells. U87 cells were infected with Lv-NC or Lv-miR-129 for 96 hours and the efficiency of miR-129 overexpression was tested by (**A**) fluorescence microscopy, (**B**) flow cytometry and (**C**) qPCR (mean ± SEM of independent experiments, $n = 3$, **$P < 0.01$), the infection efficiency of pHAGE and pHAGE-129 lentivirus was nearly 95%. (**D**) MiR-129 sponge vector was constructed to knockdown the expression of miR-129. After transfection with miR-129 sponge vector (129 sponge) or scramble control vector (SCR sponge) for 48 hours, miR-129 expression was detected by qPCR analysis (mean ± SEM of independent experiments, $n = 3$, ***$P < 0.001$).

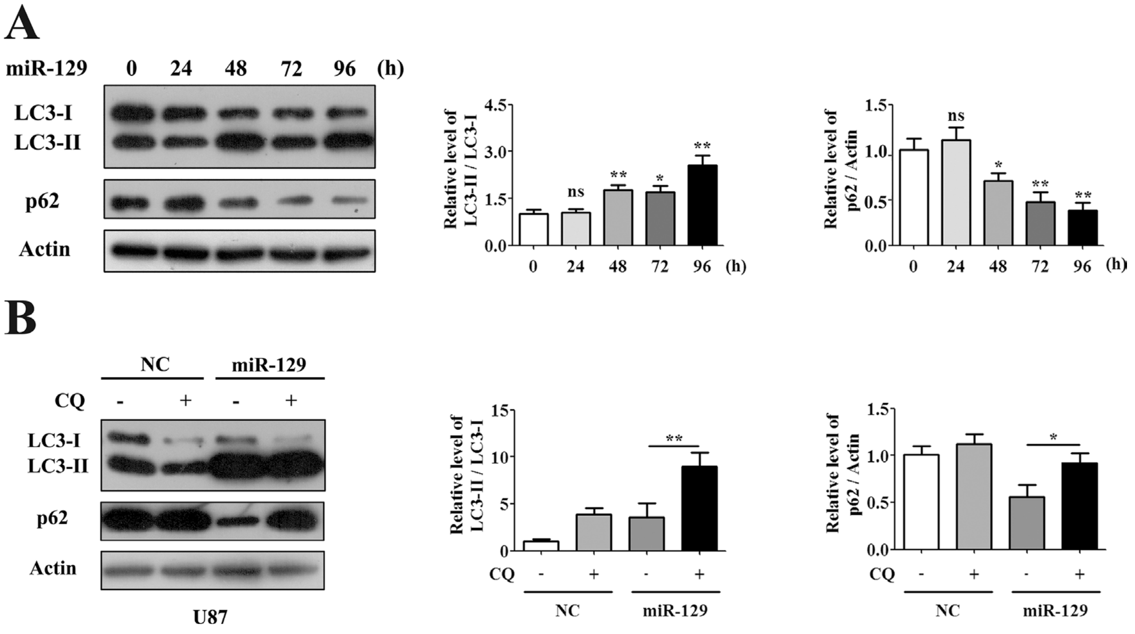

Supplementary Figure S2: Enforced expression of miR-129 induced autophagic activity. (**A**) The protein level of LC3 and p62 after infected with Lv-miR-129 for 0, 24, 48, 72 and 96 hours. The LC3-II/ LC3-I or p62/Actin ratios from immunoblots were evaluated by Image J densitometric analysis (mean ± SEM of independent experiments, $n = 3$, *$P < 0.05$, **$P < 0.01$, ns, not significant). (**B**) U87 cells were treated with or without 25 µM CQ for 6 hours after infected with Lv-NC or Lv-miR-129 for 90 hours. Cells were harvested for Western blot. The LC3-II/ LC3-I or p62/Actin ratios from immunoblots were evaluated by Image J densitometric analysis (mean ± SEM of independent experiments, $n = 3$, *$P < 0.05$, **$P < 0.01$).

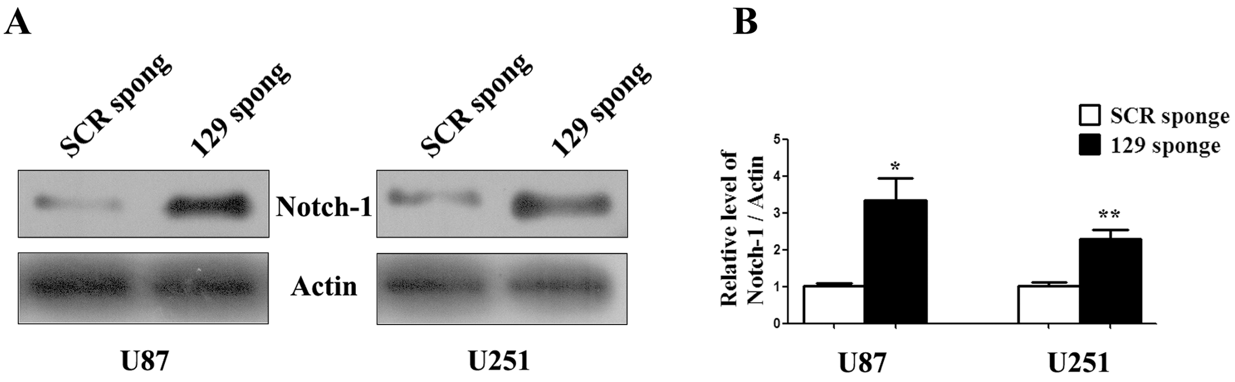

Supplementary Figure S3: The inhibition of endogenous miR-129 upregulates the protein level of Notch-1. (**A**) Notch-1 protein levels were increased after U87 or U251 cells were transfected with 129 sponge for 72 hours. (**B**) Notch-1/Actin ratios were calculated using image J densitometric analysis (mean ± SEM of independent experiments, $n = 3$, *$P < 0.05$, **$P < 0.01$).

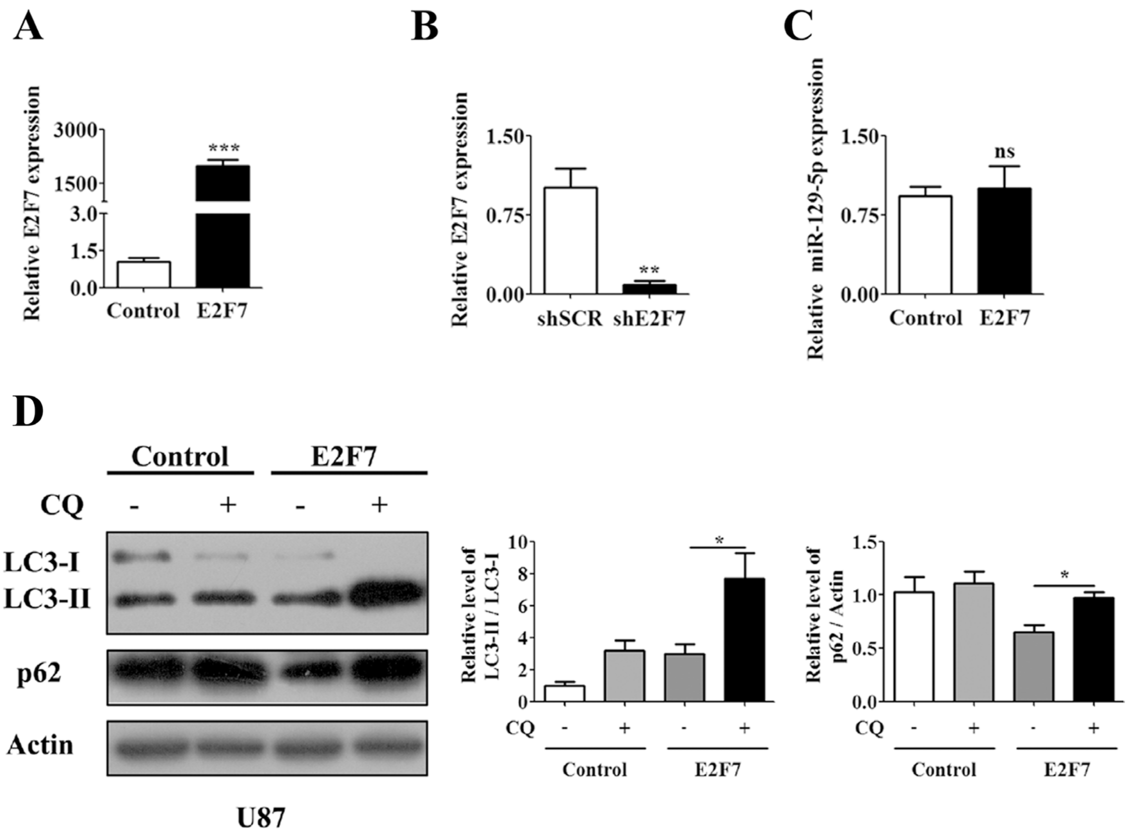

Supplementary Figure S4: Enforced expression of E2F7 increases autophagic activity. (**A**) qPCR analysis of mRNA expression levels of E2F7 after transfection with E2F7 overexpression vector or pcDNA3.1 control vector for 24 hours in U87 cells (mean ± SEM of independent experiments, $n = 3$, ***$P < 0.001$). (**B**) qPCR analysis of mRNA expression levels of E2F7 after transfection with shSCR or shE2F7 for 48 hours in U87 cells (mean ± SEM of independent experiments, $n = 3$, **$P < 0.01$). (**C**) E2F7 had no obvious effect on miR-129 expression. After transfection with E2F7 overexpression vector or pcDNA3.1 control vector for 24 hours in U87 cells, miR-129 expression was detected by qPCR analysis. (**D**) U87 cells were treated with or without 25 μM CQ for 6 hours after transfected with 3 μg pcDNA3.1 or E2F7 vector for 42 hours. Cells were harvested for Western blot. The LC3-II/ LC3-I or p62/Actin ratios from immunoblots were evaluated by Image J densitometric analysis (mean ± SEM of independent experiments, $n = 3$, *$P < 0.05$).

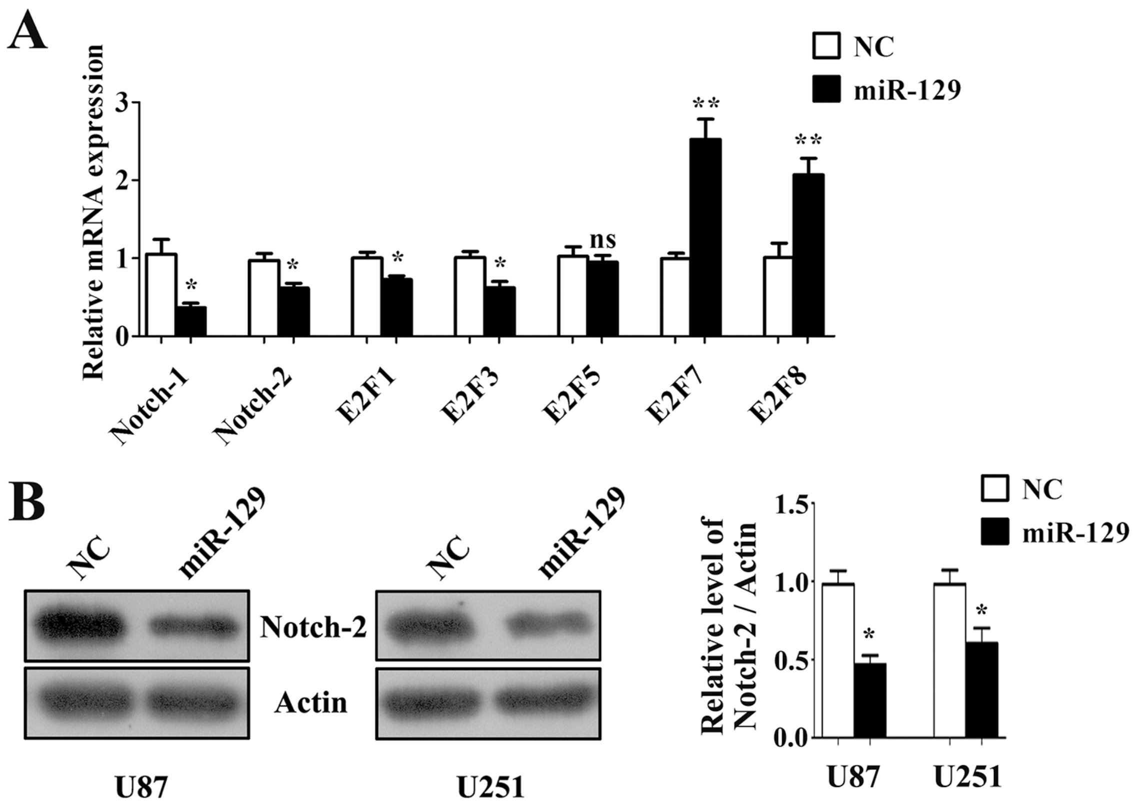

Supplementary Figure S5: MiR-129 inhibited Notch-2 expression in glioma cells. (**A**) qPCR analysis of mRNA expression levels of Notch-1, Notch-2, E2F1, E2F5, E2F7 and E2F8 after infected with Lv-NC or Lv-miR-129 for 72 hours (mean ± SEM of independent experiments, $n = 3$, **P* < 0.05, ***P* < 0.01, ns, not significant). (**B**) Western blot analysis of the protein expression level of Notch-2 after infected with Lv-NC or Lv-miR-129 for 96 hours in U87 cells (mean ± SEM of independent experiments, $n = 3$, **P* < 0.05).

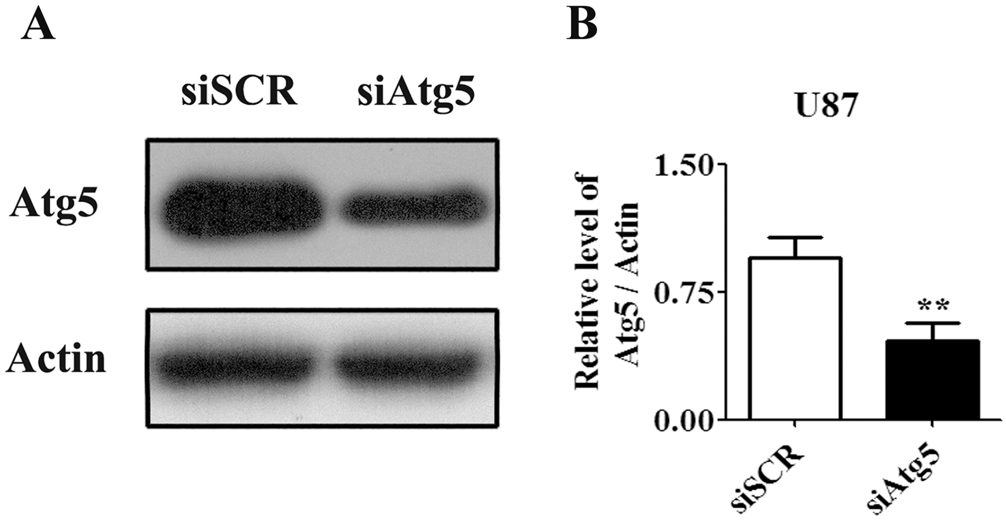

Supplementary Figure S6: The inhibition efficiency of Atg5 siRNA in U87 cells. (**A**) Western blot analysis of protein expression levels of Atg5 after transfection with 50 nM siAtg5 or siSCR for 72 hours in U87 cells. (**B**) Image J densitometric analysis of the Atg5/Actin ratios from immunoblots (mean ± SEM of independent experiments, $n = 3$, ***P* < 0.01).

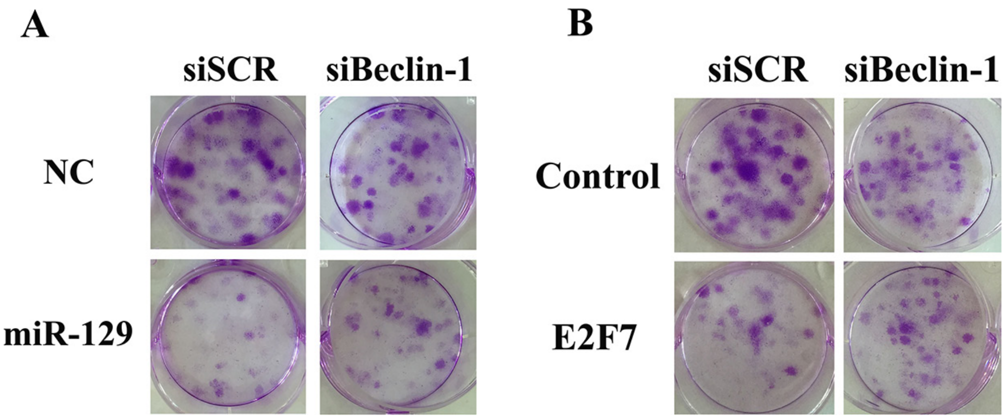

Supplementary Figure S7: Suppressed miR-129- or E2F7-induced autophagy by siBeclin-1 rescued cell proliferation. (**A**) U87 cells were transfected with 50 nM NC mimics or hsa-miR-129 mimics and 50 nM siSCR or siBeclin-1 for 6 hours. Then the cells were washed with PBS and the cell medium were changed to fresh 10% fetal bovine serum (FBS) and cultured for another 2 weeks. (**B**) U87 cells were transfected with 3 µg control or E2F7 vector and 50 nM siSCR or siBeclin-1 for 6 hours. Then the cells were washed with PBS and the cell medium were changed to fresh 10% FBS and cultured for another 2 weeks.

Table 1: Primers used for qPCR

No.	Primer name	Sequence 5′-3′
1	Notch-1-F	CGCACAAGGTGTCTTCCAG
2	Notch-1-R	AGGATCAGTGGCGTCGTG
3	Notch-2-F	GTGCCTATGTCCATCTGGATGG
4	Notch-2-R	AGACACCTGAGTGCTGGCACAA
5	E2F1-F	CATCCCAGGAGGTCACTTCTG
6	E2F1-R	GACAACAGCGGTTCTTGCTC
7	E2F3-F	GGTCCTGGATCTGAACAAGGC
8	E2F3-R	CCTTCCAGCACGTTGGTGAT
9	E2F5-F	CCATTCAGGCACCTTCTGGTAC
10	E2F5-R	AGCAGCACATGGATAGGTCCTG
11	E2F7-F	ACCCTCAGATTCCACAGACC
12	E2F7-R	AGTTTGCTGTTGCCTTTCCT
13	E2F8-F	GAGGCTCAAAGAGGGCAAGCAT
14	E2F8-R	ATGAGCACTGCGTGAGAGGGAT
15	Atg5-F	TGTGCTTCGAGATGTGTGGTT
16	Atg5-R	ACCAACGTCAAATAGCTGACTC
17	Atg7-F	CGGCGGCAAGAAATAATG
18	Atg7-R	CCCAACATCCAAGGCACTAC
19	Atg12-F	AGTAGAGCGAACACGAACCA
20	Atg12-R	GGAAGGAGCAAAGGACTGAT

21	Atg14-F	ATGAGCGTCTGGCAAATCTT
22	Atg14-R	CCCATCGTCCTGAGAGGTAA
23	BECN1-F	AGGTTGAGAAAGGCGAGACA
24	BECN1-R	TTTTGATGGAATAGGAGCCG
25	GAPDH-F	AGGTGAAGGTCGGAGTCA
26	GAPDH-R	GGTCATTGATGGCAACAA
